# Recreational Nitrous Oxide Abuse Causing Ischemic Stroke in a Young Patient: A Rare Case Report

**DOI:** 10.7759/cureus.3761

**Published:** 2018-12-21

**Authors:** Divyansh Bajaj, Ankit Agrawal, Sonali Gupta, Suryansh Bajaj

**Affiliations:** 1 Internal Medicine, Saint Vincent Medical Center, Bridgeport, USA; 2 Internal Medicine, Rutgers Robert Wood Johnson Medical School / Saint Peter's University Hospital, New Brunswick, USA; 3 Internal Medicine, Maulana Azad Medical College and Lok Nayak Hospital, New Delhi, IND

**Keywords:** nitrous oxide, ischemic stroke, hyperhomocysteinemia

## Abstract

Hyperhomocysteinemia has been associated with an increased risk of systemic atherosclerosis, cardiovascular disease, and stroke. It can result from an impaired homocysteine metabolism resulting from a dietary deficiency of folic acid, vitamin B6, and vitamin B12 or as a result of genetic predispositions due to impaired gene function involved in the methionine and homocysteine metabolism. Nitrous oxide use can also lead to hyperhomocysteinemia by irreversibly oxidizing the cobalt atom of vitamin B12, which results in an inhibition of the enzyme methionine synthase. Here, we report a rare case of a young patient who presented with acute ischemic stroke after years of recreational nitrous oxide use.

## Introduction

Hyperhomocysteinemia has been associated with an increased risk of systemic atherosclerosis, cardiovascular disease, and stroke. Hyperhomocysteinemia can result from impaired homocysteine metabolism, resulting from a dietary deficiency of folic acid, vitamin B6, vitamin B12, or as a result of genetic predispositions due to the impaired gene function involved in methionine and homocysteine metabolism. Of these, the role of MTHFR C677T gene polymorphism has been established in different models and genetic cohorts. Recently, there have been reports of hyperhomocysteinemia in postoperative patients, which could be traced to the use of nitrous oxide as an anesthetic agent [1]. Nitrous oxide use can lead to hyperhomocysteinemia by irreversibly oxidizing the cobalt atom of vitamin B12, which results in the inhibition of the enzyme methionine synthase, involved in the re-methylation of homocysteine to methionine. Another important and forgotten link is the recreational use of nitrous oxide, which is increasingly becoming popular and prevalent in the young population. To our knowledge, recreational nitrous oxide abuse resulting in ischemic stroke is rare. Here, we report the case of a young male who presented with acute ischemic stroke after five years of nitrous oxide abuse.

## Case presentation

**Figure 1:**
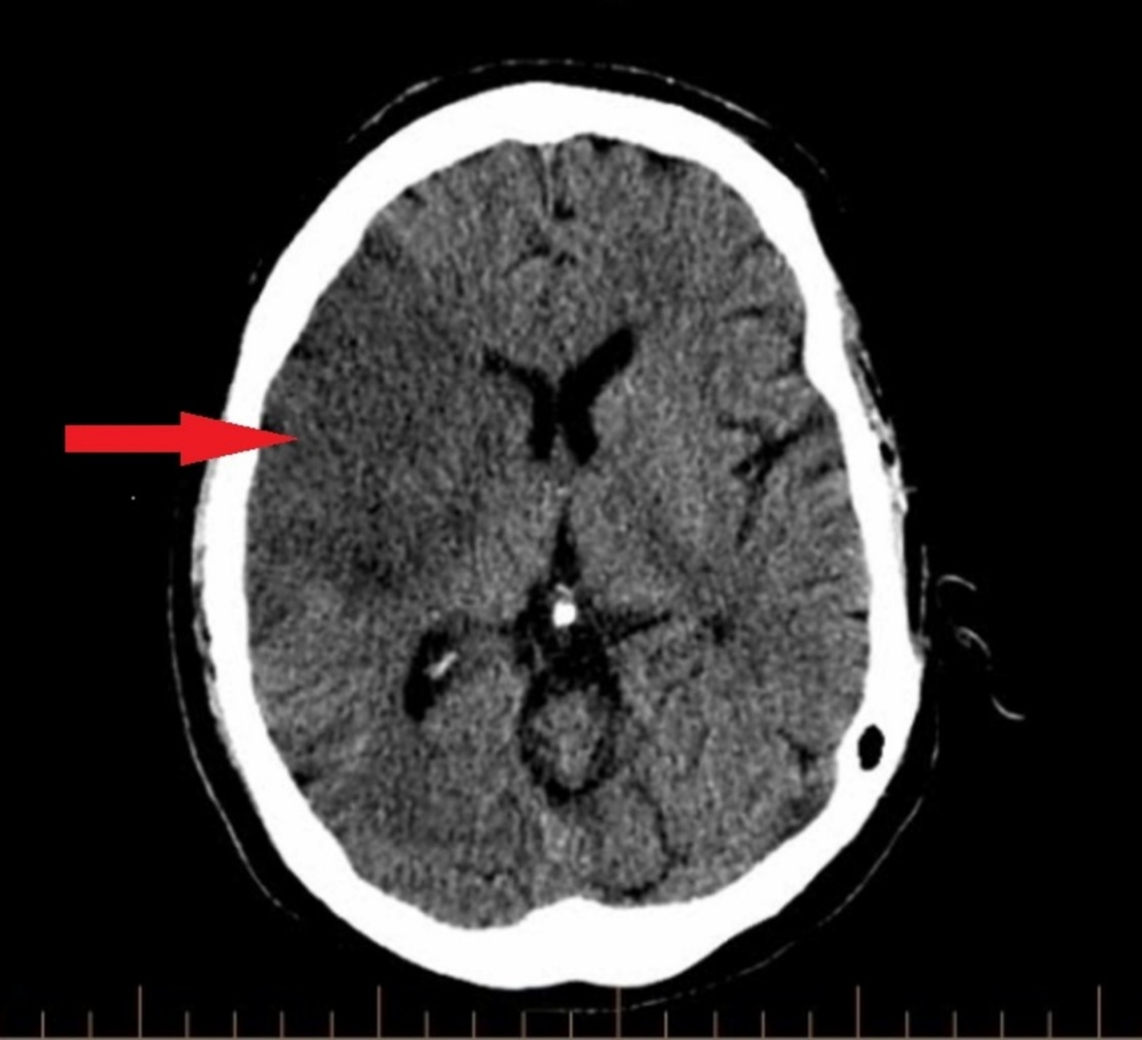
Noncontrast CT head showing a large right middle cerebral artery territory infarct involving the right frontal, right temporal, and right parietal lobes CT head demonstrating hypoattenuation of the right frontoparietotemporal cortex (red arrow) suggestive of ischemic infarct CT: computed tomography

**Figure 2:**
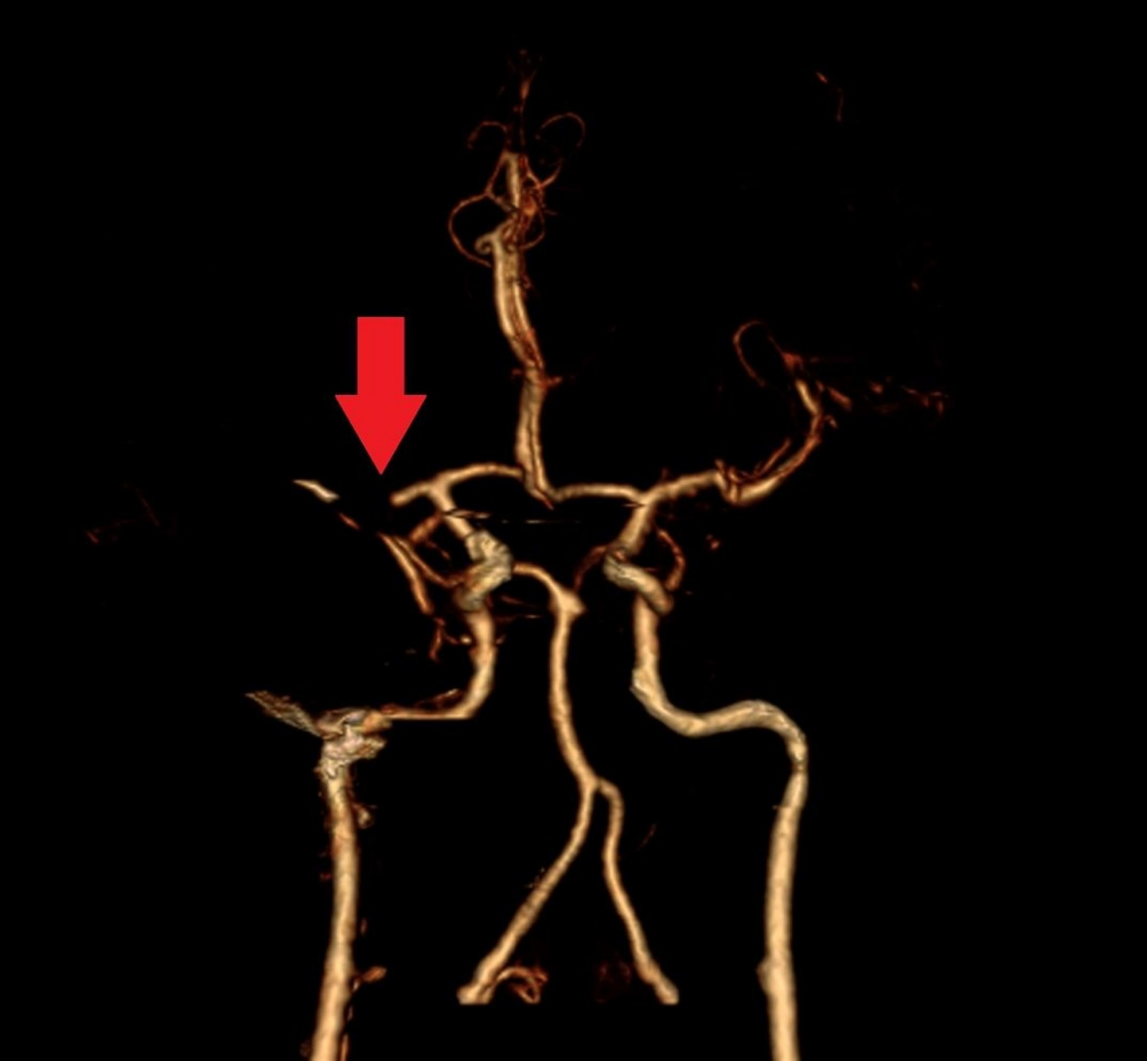
CT angiogram head showing occlusion of the right middle cerebral artery Complete occlusion of the M1 segment of the right middle cerebral artery is seen on CT angiogram (red arrow) CT: computed tomography

**Figure 3:**
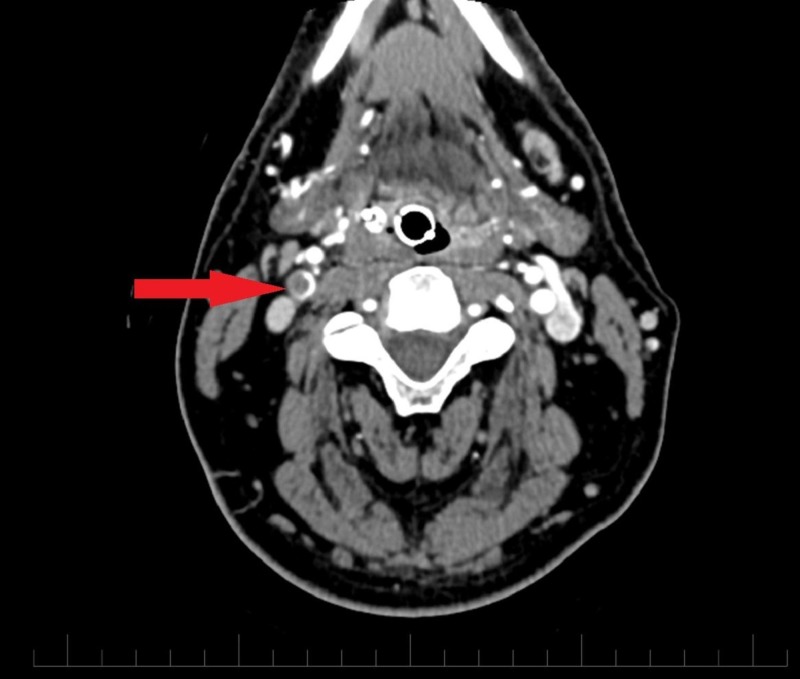
CT angiogram neck (transverse section) showing nonocclusive thrombus in the right internal carotid artery CTA neck demonstrates a hypodense filling defect (red arrow) in the right internal carotid artery suggestive of nonocclusive thrombus CTA: computed tomography angiogram

**Figure 4:**
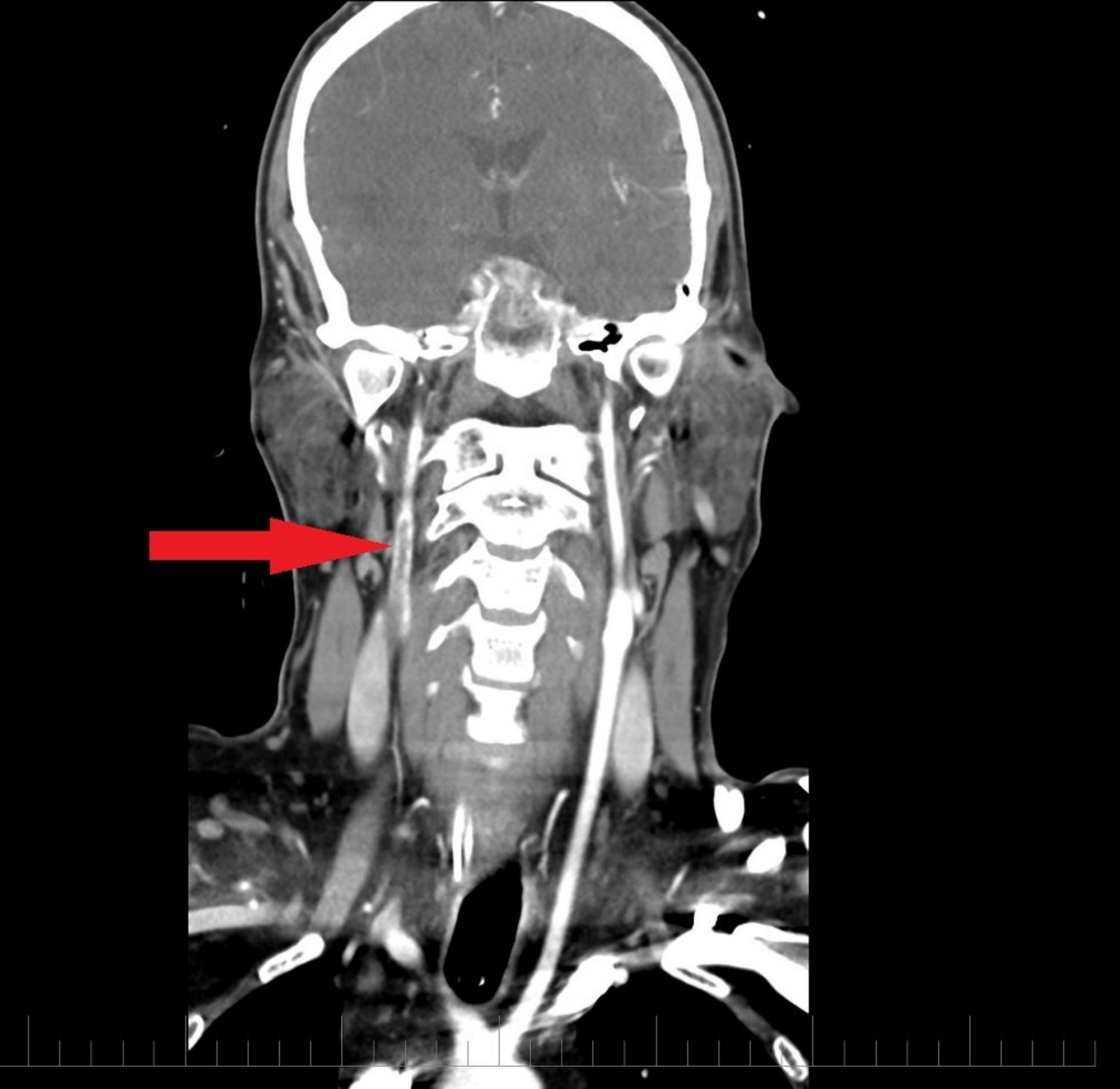
CT angiogram neck (longitudinal section) showing nonocclusive thrombus within proximal right internal carotid artery. CTA neck (longitudinal section) demonstrates a hypodense filling defect (red arrow) suggestive of nonocclusive thrombus in the proximal right internal caortid artery just distal to the bifurcation of the common carotid artery CTA: computed tomography angiogram

A 32-year-old male with a known history of bipolar disorder was brought to the emergency department with altered mental status. The patient's symptoms started with slurring of speech and left-sided motor weakness a day before the presentation. The patient suffered two episodes of seizures in the emergency department, which were controlled with two intravenous doses of lorazepam. He had no prior history of seizure disorder. He had to be immediately intubated and sedated for airway protection and was transferred to the intensive care unit from the emergency department. Noncontrast computed tomography (CT) of the head was significant for an infarct in the right frontotemporoparietal region of the brain (Figure 1). A CT angiogram of the head and neck revealed complete occlusion of the right middle cerebral artery (Figure 2) and a nonocclusive thrombus in the right internal carotid artery (Figures 3-4). The etiology of stroke was unclear at this time. There was no personal history of known thrombophilia and family history was not available, as he was an adopted child. Laboratory investigations were pertinent for macrocytic anemia (hemoglobin: 11.2 mg/dl, mean corpuscular volume: 105 fl/cell). The Factor V Leiden, protein C, and protein S levels were within normal limits. A urine toxicology test obtained prior to administering lorazepam to the patient was negative. The vitamin B12 and folate levels were found to be low (198 pg/ml, and 2.5 ng/ml, respectively). The methylmalonic acid level was in the normal range (0.12 mcmol/L; ref. range: 0.0-0.4 mcmol/L) while the homocysteine level was elevated (253 mcmol/L; ref range: 0-10). Laboratory findings were significant for hyperhomocysteinemia, which led us to gather more history in order to understand its etiology. It was ultimately revealed that the patient had been inhaling nitrous oxide as a recreational agent for the past five years. The patient's symptoms at presentation were past the 4.5 hour time window for thrombolytic therapy and, therefore, he was not a candidate for it. He was mechanically ventilated and oral aspirin 81 mg and atorvastatin 80 mg once daily was administered through an orogastric tube for secondary prophylaxis of ischemic stroke. He was also started on intravenous levetiracetam for seizure prophylaxis. On Day 5 of admission, the patient had a successful weaning trial and was subsequently extubated. Physical therapy and speech therapy were provided to the patient, and he was discharged home on Day 11 of admission. The patient continues to follow up in our neurology clinic. On his last follow-up, 12 months after discharge from the hospital, the patient had residual, left-sided motor weakness in both the upper and lower extremities and mild dysarthria without any signs and symptoms of peripheral neuropathy.

## Discussion

An increased plasma total homocysteine level is an independent risk factor for vascular events. Plasma levels above 20 μmol/L are associated with a 10-fold increase in vascular risk [2]. Various experimental and human studies have shown that increased homocysteine levels are associated with endothelial dysfunction and promote a prothrombotic state [3].

Our case reaffirms the role of increased blood N_2_O in causing hyperhomocysteinemia. The underlying metabolic mechanism is based on the fact that N_2_O acts as a potent inhibitor of methionine synthetase, such that even low concentrations of N_2_O decrease enzyme activity to undetectable levels within hours. More specifically, as a potent oxidizing agent, N_2_O acts on vitamin B12’s cobalt atom and irreversibly converts it from a 1+ to a 3+ valence state, thereby interfering with B12 bioavailability [4]. Since B12 is a necessary cofactor for methionine synthetase, as well as a number of other enzymes, the metabolism of homocysteine to methionine is significantly impaired.

The decreased activity of methionine synthetase, leading to the accumulation of homocysteine, can have serious clinical implications, including myocardial ischemia, stroke, and dementia. Elevated homocysteine levels are known to cause increased rates of thrombosis and atherosclerosis through various means, but the complete biochemical scope of these phenomena remains unclear. Sauls et al. found that direct injection of rabbits with homocysteine resulted in an acquired dysfibrinogenemia in which the higher molecular weight forms of fibrinogen that are more resistant to degradation by plasmin were detected in the animals’ serum [5]. In another vein, Hajjar et al. were able to determine the specific amino acid sequence of the binding domain of annexin-II required to complex with and activate tissue plasminogen activator (t-PA), noting that the cysteine residues it contains can complex instead with homocysteine to form disulfide bridges, thereby inhibiting normal fibrinolysis [6].

Although not exhibited in our patient, the decreased production of methionine and its metabolite S-adenosylmethionine (SAM) can additionally result in the impaired production of the myelin sheath, leading to characteristic peripheral neuropathy in a stocking-glove distribution. Lack of conversion of methionine to SAM also impairs the production of tetrahydrofolate (THF), which interferes with the normal synthesis of deoxyribonucleic acid (DNA) [4].

As clinicians may recognize, the resultant hypercoagulability and neurological dysfunction caused by nitrous oxide use may present identically to a B12 deficiency as a result of insufficient dietary intake or malabsorption. Therefore, thorough history-taking can be pivotal to uncovering the underlying pathology responsible for the clinical picture. Naturally, it is important to rule out other causes of B12 deficiency by performing a Schilling test and by measuring anti-parietal cells and anti-intrinsic factor levels. A venereal disease research laboratory test and Lyme disease titers may also be warranted to assess neuropathy. For diagnostic purposes, it is notable that patients may present with symptoms after chronic exposure to N_2_O or even after a single use. In the latter group, B12 levels may be normal with only increases in homocysteine and/or methylmalonic acid [4]. The heavy intake of nitrous oxide in the form of energy drinks could be a risk factor for hyperhomocysteinemia and, thus, thrombotic events like ischemic stroke.

Our case illustrates the previously established association between the inhaled intake of nitrous oxide and the increased risk of ischemic stroke in a young male patient.

## Conclusions

A wide spectrum of etiologies should always be considered while evaluating young patients with ischemic strokes. The recreational use of nitrous oxide can potentially cause ischemic stroke, leading to severe neurological deficits. Our case reiterates the importance of complete history taking in making an accurate diagnosis.
